# Technical Note: Left Subclavian Artery Scallop Endografts to Facilitate a Proximal Landing Zone and Upper Extremity Access for Branched Endovascular Aortic Repair of Type II Thoracoabdominal Aortic Aneurysms

**DOI:** 10.1007/s00270-021-02909-y

**Published:** 2021-07-16

**Authors:** Lydia Hanna, Ammar Abdullah, Richard Gibbs, Michael Jenkins, Mohammad Hamady

**Affiliations:** 1 Department of Surgery and Cancer, St Mary’s Hospital, Imperial College London, 10th Floor QEQM Building, St Mary’s Hospital, Praed Street, London, W21NY UK; 2 Imperial Vascular Unit, Imperial College Healthcare NHS Trust, London, UK; 3 Department of Interventional Radiology, Imperial College Healthcare NHS Trust, London, UK

**Keywords:** Scallop thoracic endovascular aortic repair, Left subclavian artery, Branched endovascular aortic repair, Upper limb access

## Abstract

**Purpose:**

To describe the dual purpose of left subclavian artery (LSA) scallop endografts to create the proximal landing zone (PLZ) *and* facilitate antegrade left-sided upper extremity access for branched endovascular aortic repair (BEVAR) of Type II thoracoabdominal aneurysms (TAAA) with a short PLZ.

**Technique:**

Three patients with an inadequate (< 20 mm) PLZ underwent a 2-stage repair of Type II TAAA. Following femoral cut-down, a custom-made LSA scallop endograft was deployed into zone 2 to create the PLZ and maintain perfusion to the LSA. In a second procedure 36–96 days after insertion of the scalloped thoracic stent-graft, a branched abdominal stent-graft was subsequently deployed to dock into the proximal scallop endograft as the second stage. Via a left axillary conduit, a 12Fr sheath was used to cannulate the LSA scallop to facilitate selective catheterisation of antegrade branch cuffs and renovisceral target vessels, and insertion and deployment of bridging stents. The LSA scallop was also used to selectively catheterise and stent the perfusion branches via left-sided brachial puncture that were left open in each of the three cases 8–14 days after the second procedure to minimise the risk of spinal cord ischaemia. There were no neurological or endoleak complications.

**Conclusion:**

LSA scallop endografts are a feasible and useful adjunct to create the PLZ and to provide antegrade access for visceral stenting of branches and target vessels through the LSA scallop in branched endovascular repair of Type II TAAA with short PLZ.

## Introduction

Longitudinally orientated renovisceral vessels that originate from an aneurysmal aortic lumen, as in type II thoracoabdominal aneurysms (TAAA), require upper extremity access to facilitate cannulation of downward-orientated (antegrade) branch cuffs [1–3]. The left side is preferable to the right given the increased risk of cerebrovascular events [4]. Extensive TAAA disease up to the left subclavian artery (LSA) presents a challenge to the suitability of the proximal landing zone (PLZ). Coverage of the LSA with/without the use of left carotid artery-to-left subclavian artery bypass facilitates creation of an adequate PLZ but renders left-sided upper extremity access impossible. We describe the dual purpose of the Relay Scallop (Terumo Aorta, Sunrise, Florida, USA) endograft to the LSA for the creation of a zone 2 PLZ *and* preservation of left-sided upper extremity access for BEVAR of Type II TAAAs.

## Technique

**Fig. 1:**
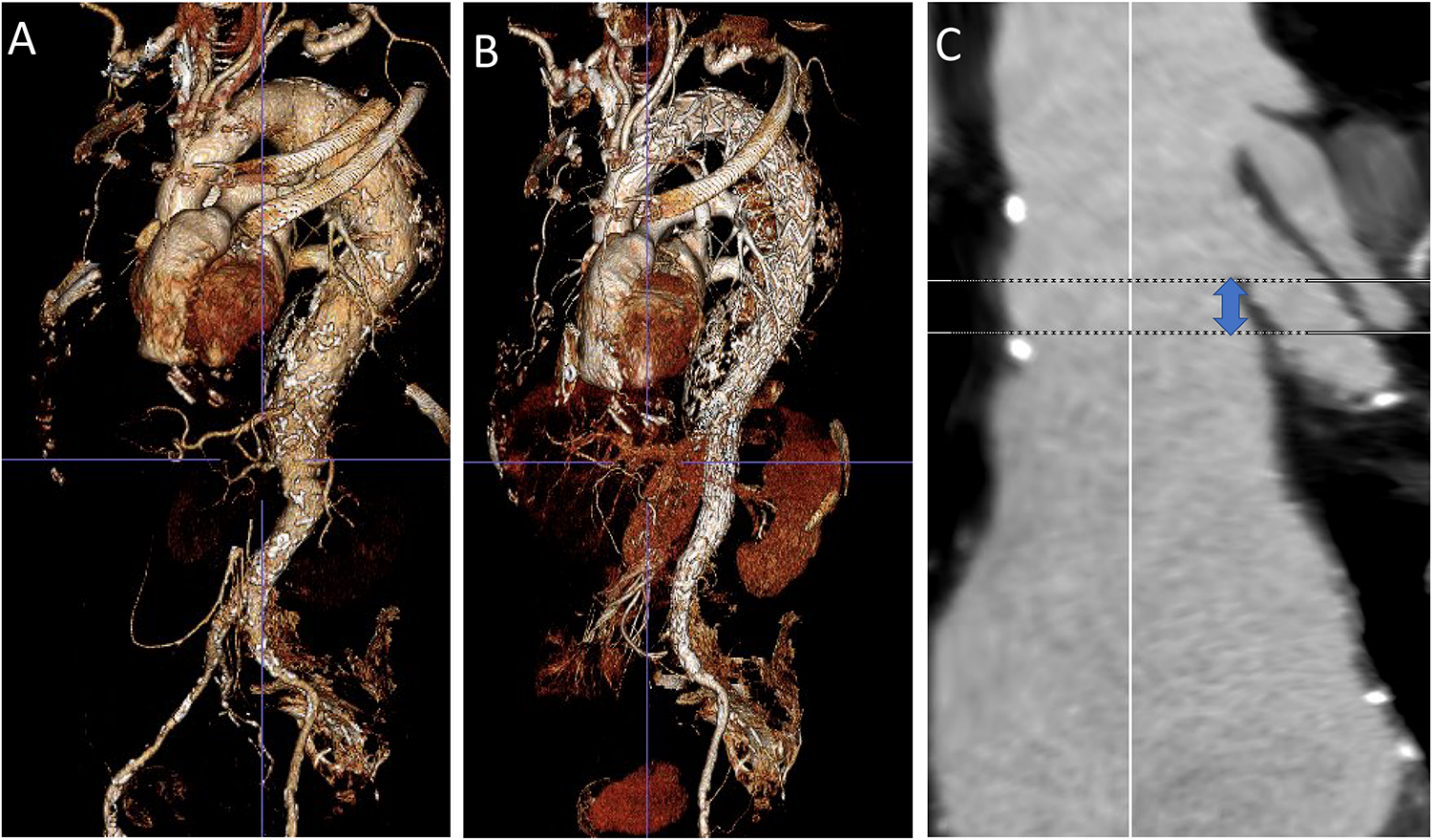
Preoperative volume rendered reconstruction of a Type II thoracoabdominal aortic aneurysm (**a**), and postoperative volume rendered reconstruction following two-stage endovascular repair (**b**) and centreline reconstruction of the length of proximal landing zone (distance between distal edge of LSA and start of the pathology that was 8 mm in this patient) (C)

Three male patients between 69 and 78 years and ASA 3 with Crawford Type II atherosclerotic TAAA (mean aneurysm diameter 6.5 cm (range, 5.9–7.2 cm) and a PLZ < 20 mm from the LSA (patient 1: 6 mm; patient 2: 8 mm; patient 3: 11 mm; Fig. 1)) underwent a two-stage endovascular repair comprising a custom-made Relay proximal scallop thoracic endograft to the LSA (Terumo Aorta, Sunrise, Florida, USA) and a custom-made branched endograft (Cook. Medical, Bloomington, Ind) involving 4 antegrade branch cuffs. Stent-graft specifications are detailed in Table 1.

**Table 1:** Relay Scallop endograft specifications for the three patients

	**Main body size** (proximal diameter-distal diameter-length, mm)	**Scallop dimensions** (diameter-length, mm)
Patient 1	36-30-200	19-27
Patient 2	36-28-185	19-22
	34-28-155	
Patient 3	34-30-200	18-30

All cases were performed in a hybrid operating suite under general anaesthesia and systemic heparinisation to achieve an activated clotting time > 250 s. To minimise the risk of spinal cord ischaemia from Type II TAAA repair, we employ cerebrospinal fluid drainage in all BEVAR procedures as well as temporary aortic sac perfusion with perfusion branches. The perfusion branch that is left open is usually the one that has a downward facing target vessel, facilitating cannulation at the final stage.

**Fig. 2:**
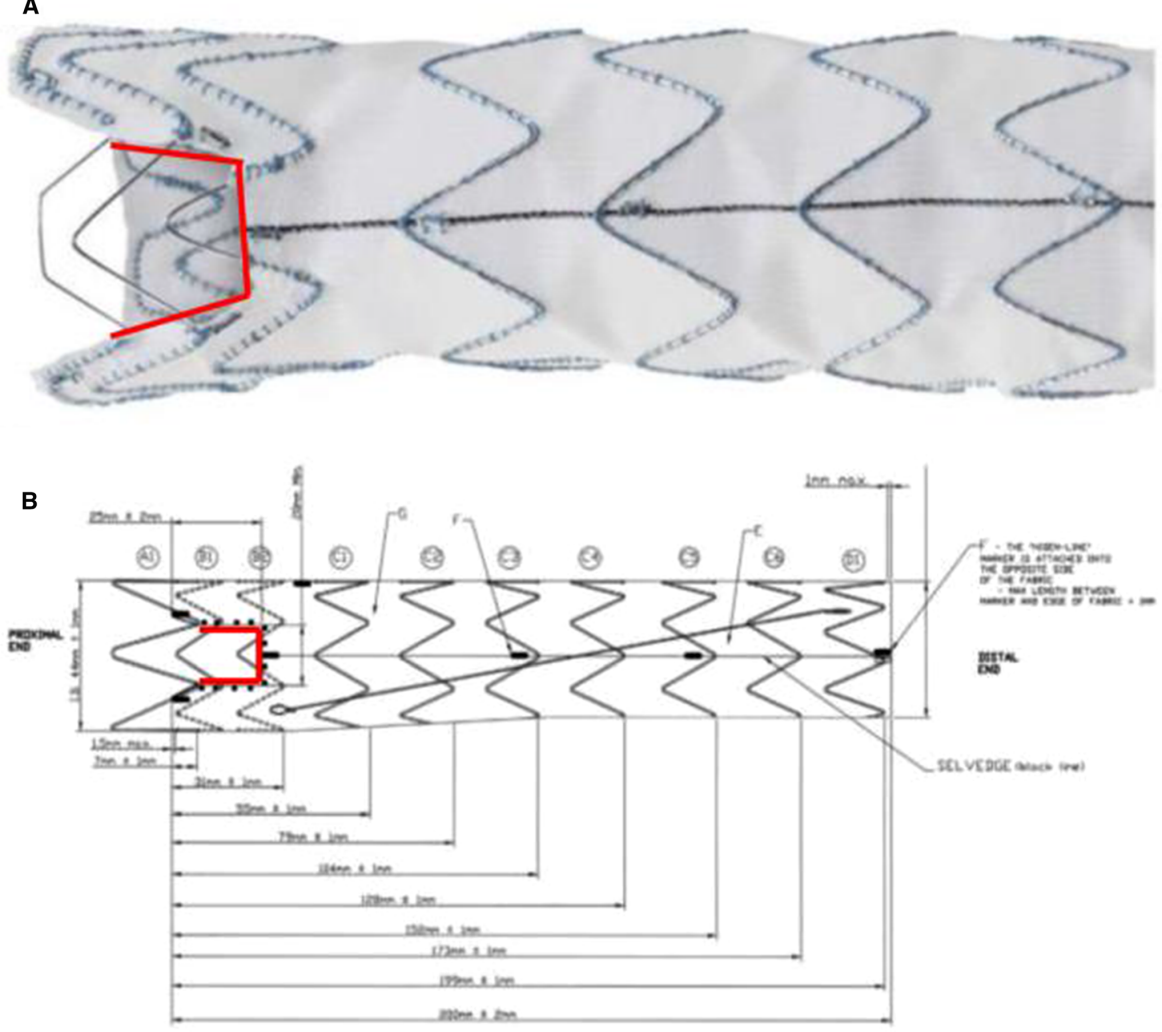
Image (**a**) and diagrammatical representation (**b**) of the scalloped endograft with the radiopaque markers outlined

The Relay proximal scallop thoracic endograft and delivery system is based upon the Relay platform and has been previously described [5–7]. The scallop is delineated by four radiopaque markers to be aligned with the origin of the LSA. The width of the scallop is the diameter of the LSA plus 2 mm and must not exceed > 50% of the endograft diameter. The landing zone incorporates the vessel to be scalloped (Fig. 2).

**Fig. 3:**
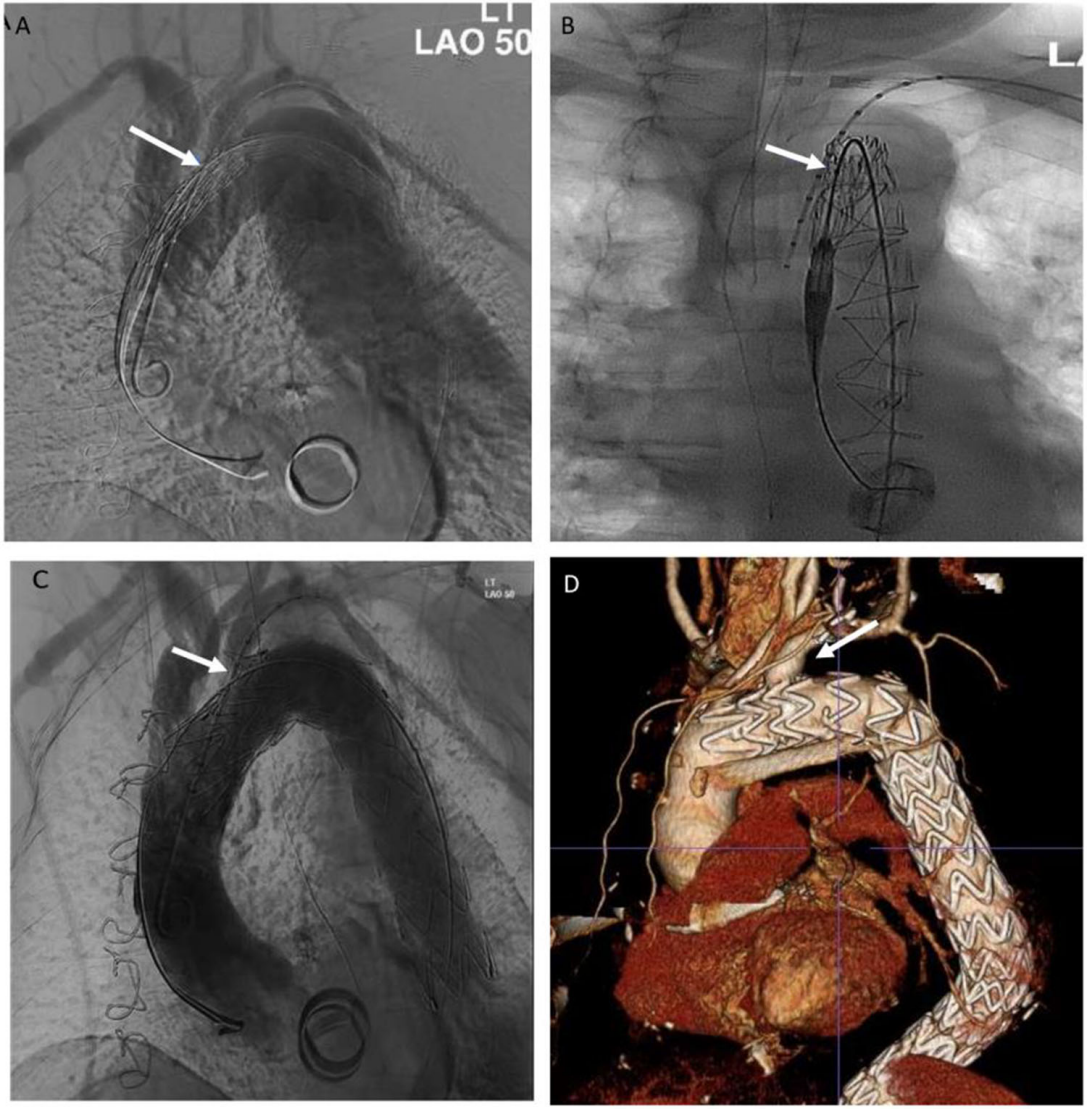
Perpendicular aortic arch angiograms showing radiopaque markers of scallop (white arrows) to left subclavian artery (LSA) with the undeployed (**a**, **b**) and deployed (**c**) endograft with perfusion to the LSA and no type I endoleak intraoperatively at completion angiogram (**c**) and at latest follow-up on computed tomography (**d**)

Following femoral cut-down the Relay system was advanced into the mid-descending thoracic aorta, followed by further advancement of the secondary sheath into the aortic arch over a 0.035-inch Meier wire (Boston Scientific, Massachusetts). A pigtail catheter was introduced into the aortic arch for angiographic LSA identification through a left-sided percutaneous brachial puncture. Two angiograms at perpendicular angles were obtained to confirm alignment of the scallop radiopaque markers to the ostium of the LSA (Fig. 3). Following pharmacological reduction of the systolic blood pressure to 70 mmHg, the endograft was deployed 5–10 mm above the coeliac axis.

All patients returned for BEVAR 36–96 days after the first procedure; bilateral femoral cut-downs were used for insertion of the branched and bifurcated endografts. A left axillary conduit was fashioned for the insertion of bridging renovisceral covered stents.

**Fig. 4:**
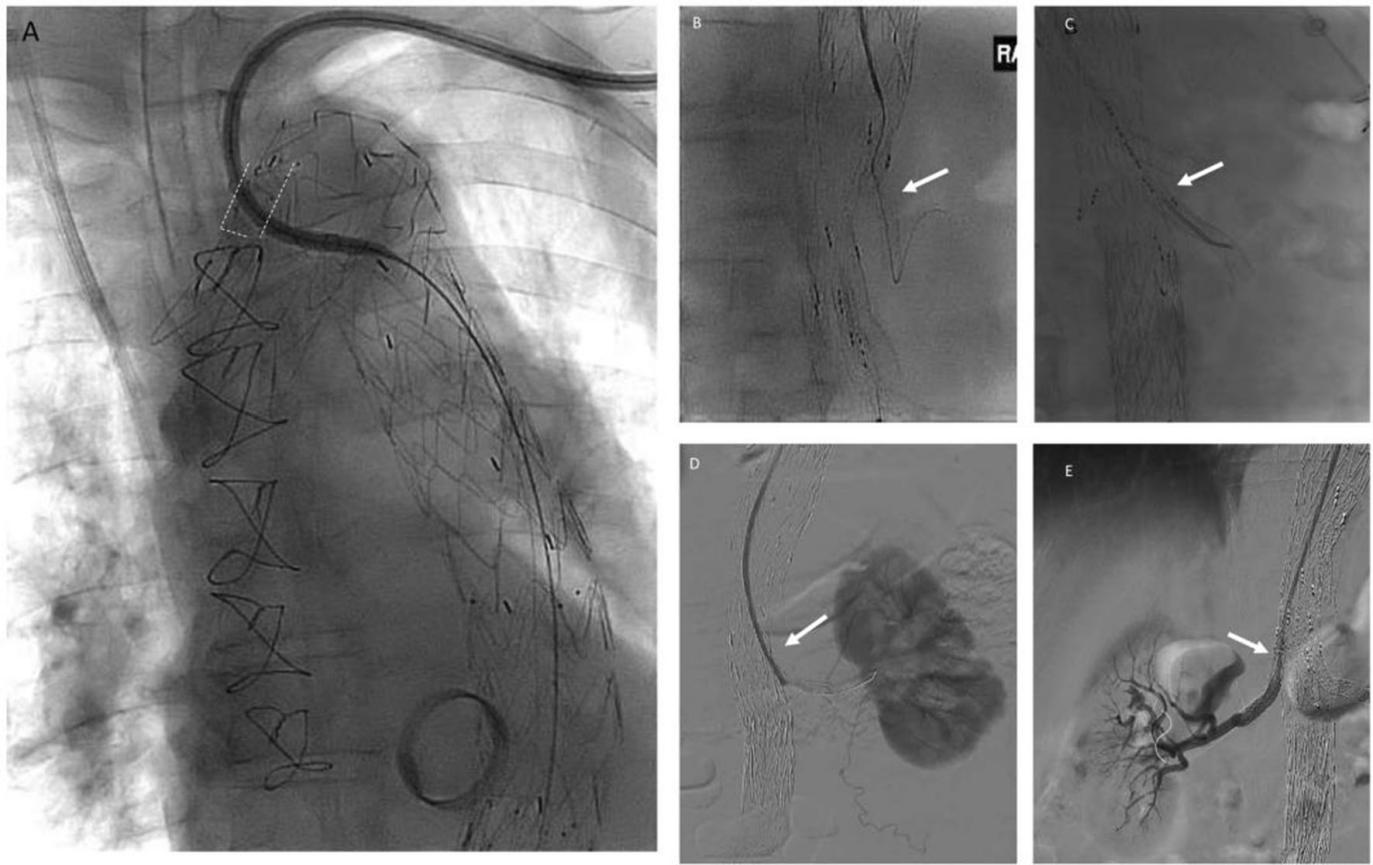
Left-sided upper extremity access. Angiograms showing selective catheterisation of LSA scallop and introduction of Ansel catheter into descending aorta (radiopaque markers outlined with white dashed line, **a**) to facilitate catheterisation of antegrade target vessels (white arrows; **b** coeliac artery, **c** superior mesenteric artery **d** left renal artery, **e** right renal artery)

Once the branched endograft was inserted and deployed, the iliac limbs were inserted and deployed. The femoral sheaths were removed, and the femoral arteriotomies were closed with interrupted 5-0 prolene for early pelvic and lower limb perfusion. The axillary conduit was then accessed, and a 12F Ansel sheath (Cook Medical) was advanced into the descending thoracic aorta via LSA scallop. Each side branch cuff and target vessel were catheterised, and appropriately sized bridging covered stent was deployed (Fig. 4). A total of 9 renovisceral vessels were successfully stented, and a perfusion branch was left open in each patient (coeliac, right renal, left renal).

During the same admission (8–14 days following the second procedure), perfusion branch stents were successfully introduced via the LSA scallop and mated with the respective target vessels and branch cuffs via a left brachial puncture and insertion of a 7Fr sheath. There were no cases of periprocedural stroke or type I/III endoleaks, wound or other complications from the axillary conduit or left brachial puncture. The LSA remains perfused via the scallop in all three patients at most recent follow-up (median of 2 years).

## Discussion

The Relay LSA proximal scallop endograft permits extension of the PLZ into zone 2 achieving a 20 mm seal zone length across the inner aortic curve for proximal aneurysm exclusion, while the ‘u’-shaped defect (scallop) in the upper part of the proximal edge of the endograft fabric maintains antegrade perfusion to the LSA. This obviates the need for extra-anatomical bypass procedures, reducing surgical risk for patients [8] and simultaneously preserves left upper extremity access for cannulation of the renovisceral vessels during BEVAR. To our knowledge, this is the first report to describe the use of scallop endografts for this dual purpose. The deployment steps are the same as for standard Relay devices, making this device widely applicable [5].

Several European centres have published the technical success of this endograft when the seal zone is < 20 mm from the LSA as a result of extensive disease, or in angulated aortic arches that limit stent-graft apposition at the inner aortic curvature [5–7]. In our own experience of 19 patients treated with a Relay LSA scallop endograft, there were no major strokes and was only 1 minor stroke [5]. Only 1 patient experienced a type Ia endoleak requiring intervention 2 years after the index procedure (unpublished data). While there is no ‘IFU’ as these are custom-made devices, absolute exclusion criteria, according to our experience, include > 90° arch angulation at the LSA, < 5 mm distance between the start of the pathology and distal edge of the target supra-aortic vessel (risk of type 1a endoleak from the scallop), < 5 mm distance between the supra-aortic trunks, width of the targeted supra-aortic vessel is > 50% of the diameter of the endograft (risk of endograft integrity). The three-week manufacturing time precludes emergent use.

The alternative approach to this technique would have been LSA coverage with/without revascularisation and right-sided upper extremity access. LSA coverage can lead to ischaemic posterior circulation stroke due to low-flow ischaemia in the left vertebral artery [4, 8–10]. Right-sided upper extremity access increases the ischaemic stroke risk due to cerebral embolisation of atherosclerotic debris from wire and catheter manipulations across the arch and supra-aortic trunks, and cerebral hypoperfusion from partial occlusion of sheaths across the supra-aortic vessels [4].

Two recent meta-analyses have affirmed the increased stroke risk with LSA coverage with/without revascularisation, and with right-sided upper extremity access during endovascular procedures [4, 10]. Ischaemic stroke may potentially be mitigated with our described technique by maintaining perfusion to the posterior circulation territory and minimising instrument manipulation within the arch. Left-sided upper extremity access may also improve ergonomics given the reduced working distance to the renovisceral vessels, in comparison with right-sided upper extremity access.

Transfemoral techniques for retrograde cannulation with steerable sheaths, through-and-through guidewires/sutures have been described and may also avoid the potential risk of cerebrovascular events with upper extremity access [11–13]. While technically feasible, the prolonged use of large access sheaths in the femoral arteries, particularly in patients with significant iliac tortuosity to gain better stability, pushability and torqueability, may increase the risk of pelvic and lower limb ischaemia, and even spinal cord ischaemia [13, 14]. A particular advantage of our technique is that once the iliac limbs are deployed, the femoral sheaths are removed, and distal perfusion is restored promptly, allowing the operator to focus on renovisceral cannulation via left-sided upper extremity access.

## Conclusion

The use of the Relay LSA scallop endografts can be used to create a PLZ and simultaneously preserves left-sided upper extremity access for antegrade delivery of bridging stents for branched endovascular aortic repair (BEVAR) of Type II thoracoabdominal aneurysms (TAAA).
